# Cancer-Associated Thrombosis in Cirrhotic Patients with Hepatocellular Carcinoma

**DOI:** 10.3390/cancers10110450

**Published:** 2018-11-16

**Authors:** Alberto Zanetto, Elena Campello, Luca Spiezia, Patrizia Burra, Paolo Simioni, Francesco Paolo Russo

**Affiliations:** 1 Gastroenterology and Multivisceral Transplant Unit, Department of Surgery, Oncology and Gastroenterology, Padua University Hospital, 35128 Padua, Italy; alberto.zanetto@yahoo.it (A.Z.); burra@unipd.it (P.B.); francescopaolo.russo@unipd.it (F.P.R.); 2 Thrombotic and Hemorrhagic Diseases Unit, Department of Medicine, Padua University Hospital, 35128 Padua, Italy; elena.campello83@gmail.com (E.C.); luca.spiezia@unipd.it (L.S.)

**Keywords:** cancer, hepatocellular carcinoma, hypercoagulability, portal vein thrombosis, venous thromboembolism

## Abstract

It is common knowledge that cancer patients are more prone to develop venous thromboembolic complications (VTE). It is therefore not surprising that patients with hepatocellular carcinoma (HCC) present with a significant risk of VTE, with the portal vein being the most frequent site (PVT). However, patients with HCC are peculiar as both cancer and liver cirrhosis are conditions that can perturb the hemostatic balance towards a prothrombotic state. Because HCC-related hypercoagulability is not clarified at all, the aim of the present review is to summarize the currently available knowledge on epidemiology and pathogenesis of non-malignant thrombotic complications in patients with liver cirrhosis and HCC. They are at increased risk to develop both PVT and non-splanchnic VTE, indicating that both local and systemic factors can foster the development of site-specific thrombosis. Recent studies have suggested multiple and often interrelated mechanisms through which HCC can tip the hemostatic balance of liver cirrhosis towards hypercoagulability. Described mechanisms include increased fibrinogen concentration/polymerization, thrombocytosis, and release of tissue factor-expressing extracellular vesicles. Currently, there are no specific guidelines on the use of thromboprophylaxis in this unique population. There is the urgent need of prospective studies assessing which patients have the highest prothrombotic profile and would therefore benefit from early thromboprophylaxis.

## 1. Introduction

Patients with cancer are more prone to develop venous thromboembolic complications (VTE), presumably due to overexpression of tissue factors, increased platelet activation by cancer pro-coagulant proteins, and oversecretion of cytokines [1]. Additional factors such as chemotherapy, hormonal therapy, and indwelling central venous catheters can also increase the risk of VTE [2]. Similarly, cancer-related surgery is associated with a higher risk of VTE than other surgical procedures [3,4]. 

Unsurprisingly, hepatocellular carcinoma (HCC) is also associated with an increased risk of VTE, with the portal vein being the most frequent site of thrombosis (PVT), with a reported incidence of 20–40% [5]. To understand how the presence of HCC can perturb the hemostatic balance, the context in which HCC develop must be considered, with this cancer almost always being a complication of liver cirrhosis [6], with the prevalence of cirrhosis among patients with HCC estimated of 85–95% [7]. The rebalanced and unstable hemostatic status of liver cirrhosis can be easily tipped towards thrombotic complications by superimposed conditions, including HCC [8]. Additionally, cirrhotic patients may develop non-splanchnic thrombosis too, and it is ever clearer that patients with cirrhosis are not auto-anticoagulated, as previously thought, but are actually more prone to develop thrombotic complications than non-cirrhotic patients [9]. HCC may develop in non-cirrhotic liver. According to a very recent review by Kulik L. et al. [10], the occurrence of HCC without prior cirrhosis is unusual and specifically about 15% of HBV-related cases. HBV is known to be involved directly in liver mutagenesis. Additionally, among new HCC cases without advanced fibrosis or cirrhosis in the United States, non-alcoholic fatty liver disease (NAFLD) and metabolic syndrome accounted for the majority of cases [11].

Both local and systemic factors can foster the development of site-specific thrombosis, particularly PVT. Among these risk factors, the importance of HCC has long been recognized. Even though, the pathophysiology of HCC-associated hypercoagulable state has only recently been studied in depth.

The present review aims to summarise the currently available knowledge on the epidemiology and pathogenesis of non-malignant thrombotic complications in patients with liver cirrhosis and HCC. 

## 2. Thromboembolic Complications in Cirrhotic Patients with Hepatocellular Carcinoma

### 2.1. Portal Vein Thrombosis

PVT is the most common thrombotic complication in cirrhotic patients with HCC, with a 1-year incidence ranging from 7.4% up to 24% [12,13,14,15,16,17,18,19,20]. The diagnosis of PVT in patients with cirrhosis is usually made during routine ultrasound in asymptomatic patients or following a new event of hepatic decompensation. Splanchnic Doppler ultrasound is the first line method used, with a sensitivity of about 90% for complete PVT, which decreases to about 50% for partial thrombosis [9]. Additionally, a CT scan or MRI can better define the extension of PVT [21]. It is worth mentioning that patients with cirrhosis and HCC may also experience the invasion of the portal vein by neoplastic cells, as previously reported [22,23,24]. This condition, named “malignant PVT”, has a completely different pathophysiology, treatment, and prognosis [25]. Therefore, from a clinical perspective, it is crucial to establish a differential diagnosis between malignant and non-malignant thrombosis. It is generally accepted that HCC infiltration of the portal vein represents an exclusion criteria for liver transplant (LT), surgical resection, chemoembolisation, and imaging-guided ablation [25,26]. The following radiological criteria can be used to exclude the presence of malignant PVT: proximity of HCC and PVT, and enlargement of the portal vein diameter > 23 mm [27]; enhancement of the thrombus in the arterial phase of contrast injection at CT scan or contrast ultrasound [28]; or an arterial-like flow observed on Doppler ultrasound [29]. However, few cases may require a cytological analysis of the thrombus, which has proven effective for the purpose of this differential diagnosis despite been an invasive technique and relatively unsafe in patients with cirrhosis [30,31]. Here, we focused solely on the epidemiology and physiopathology of non-malignant PVT

It was first reported in the early 1990s by Nonami et al. [32] that, notwithstanding the absence of neoplastic thrombi, HCC constituted an additional risk factor for PVT development. In their landmark study conducted on an autoptic population, 34.8% of HCC patients versus 11.4% of patients without HCC experienced PVT (*p* < 0.001). Interestingly, patients with HCC showed a greater prevalence not only of intra-hepatic but also extra-hepatic (partial and complete) thrombosis than non-HCC patients. A few years later, Davidson et al. [33] confirmed these findings in a clinical setting. HCC patients who underwent LT showed a significantly higher incidence of PVT than the counterparts without liver malignancy (6/22 vs. 10/110, *p* < 0.05). The higher incidence of PVT in LT recipients listed for HCC was explained as follows: the procoagulant state induced by the malignancy itself; the study was conducted in a tertiary centre with expertise in PVT management; most HCC patients had received a number of percutaneous treatments, that can be associated with PVT development. In a more recent and similar study, Ravaioli and colleagues analysed the prevalence of PVT in a series of LT recipients [34]. In this study, PVT was present in 40.8% of recipients with HCC versus 30.7% of recipients without HCC (*p* = 0.05). In line with previous findings, patients with HCC presented a greater risk not only for PVT per se, but also for widespread pathological thrombosis (Table 1). However, it is worth mentioning that PVT is not a “yes or no” phenomenon, with its severity being correlated with the extent of the thrombosis, especially in LT candidates. Indeed, a recently updated meta-analysis observed a significant drop in survival rate in LT recipients when PVT was complete and/or the superior mesenteric vein was involved [35].

To further assess the relationship between HCC and PVT risk, we recently conducted a clinical prospective study in which we compared the incidence of PVT in patients with and without HCC, followed for 1 year and matched according to the severity of baseline liver disease. In our cohorts, the incidence of PVT more than doubled among cancer patients compared to cirrhotic patients without neoplastic diseases (24.4% [10/41] vs. 11.4% [4/35]; *p* = 0.05) [19]. Interestingly, in presence of HCC, the risk of developing PVT was much more frequent in patients with well-compensated liver disease (Child’s A cirrhosis). It is indeed well known that in cirrhotic patients, the more advanced the severity of baseline liver disease (Child’s C class; previous treatments for portal hypertension; presence of porto-systemic shunts; decompensated liver disease), the greater the coagulation imbalance and therefore the risk of PVT [24]. Thus, HCC was firstly recognized a specific risk factor in patients with less advanced liver disease, suggesting that also these patients should be considered at risk of thrombosis. Whether the etiology of baseline liver disease may influence the PVT risk in patients with cirrhosis and HCC remains unclear due to insufficient data.

### 2.2. Non-Splanchnic Venous Thromboembolism

Liver cancer is associated with a higher risk of developing non-splanchnic thrombotic complications as well. Particularly, the rate of VTE complications has been evaluated in three large scale studies (Table 1). In 1999, Levitan N. et al. [36] considered 22,938 patients with liver cancer using Medicare claims data and reported an incidence rate of VTE of 69 per 10,000 patients. The rate was intermediate between the malignancies with very high risk of VTE (kidney, stomach, pancreas, brain, ovary, and lymphoma) that showed an incidence rate of about 80–120 per 10,000 patients and those with low risk of VTE (head/neck, bladder, breast, esophagus, uterus) that showed an incidence rate of 16–50 per 10,000 patients. A subsequent study by Wun and White in 2009 showed that the incidence of VTE within one year of diagnosis of liver cancer was 1.7%, confirming an intermediate risk of liver cancer albeit comparable to that of lung cancer [37]. Finally, a population-based cohort study by Cronin-Fenton et al. in 2010 reported a cumulative incidence of 1.1% during 1-year follow-up in 550 patients with liver cancer [38]. The concomitant incidence in the general population was 0.4%. Interestingly, the incidence rates of VTE were highest for patients with pancreas, liver, lung, ovary and brain cancers. Moreover, in the same study the Authors reported a high incidence rate of hospitalization for VTE among liver cancer patients (20.4 (9.2–45.3) per 1000 person-years). Unfortunately, these studies did not distinguish between cirrhotic and non-cirrhotic HCC patients. It is entirely plausible that the high hospitalization rate might be due to the concomitant presence of cirrhosis.

Cirrhotic patients present per se a higher risk of developing non-splanchnic thrombotic complications [39] (Table 2). The association between liver cirrhosis and deep vein thrombosis (DVT) and/or pulmonary embolisms (PE) was retrospectively evaluated in case control studies which revealed that 0.8–7% of hospitalized cirrhotic patients developed VTE [39,40,41,42,43,44,45,46] (Table 2). The patients did not present a lower risk of VTE complications when compared to acute medical patients without liver disease [39,47] and, importantly, a prolonged INR had no bearing whatsoever on said risk [48]. In two landmark studies, low levels of albumin were an independent predictor of VTE at multivariate analysis, possibly mirroring the low levels of endogenous anticoagulants typically found in cirrhotic coagulopathy [39,47]. Conversely, traditional markers of coagulation impairment in liver disease (e.g., INR; platelet count) were not predictive of VTE [47] and therefore ought not to be considered in clinical practice.

What is the role of liver cancer in this setting? VTE is a common complication of malignancy [2,49,50] with varying rates among different cancers [36,51,52,53,54] and especially high rates in gastro-intestinal malignancies [51,52]. In this regard, HCC poses a unique challenge with regard to cancer-associated thrombosis owing to the underlying liver cirrhosis; hence why the prevalence and significance of VTE in cirrhotic patients with HCC are not fully understood to date.

On one hand, it would appear that HCC can be associated with an increased risk of VTE. In fact, a recent retrospective analysis evaluated HCC-related thrombophilia in a cohort of 270 cirrhotic patients with HCC [55] and uncovered a 2-year cumulative incidence of VTE after HCC diagnosis of 5.93% (16 VTEs including 7 PEs, 5 intra-abdominal DVTs, and 4 peripheral DVTs), in line with previous reports on other types of cancers [56]. Furthermore, most cases (12/16, 75%) occurred within 3 months of the HCC diagnosis (early VTE), suggesting HCC may have tipped the hemostatic balance towards a hypercoagulable state. Notably, at multivariate analysis, independent risk factors for VTE were characterized as cirrhosis-related (severity of liver disease, HR = 16.54 Child B vs. Child A; *p* = 0.015); HCC-related (HR = 3.58 over three intra-hepatic lesions vs a single lesion; *p* = 0.048); multi-organ extra-hepatic metastasis (HR = 11.94; *p* = 0.028); and non-liver related (obesity (HR = 5.56, *p* = 0.030)). As expected, the pathophysiology of VTE in HCC patients is multifactorial. The presence and biological behavior of HCC (e.g., number of nodules; extra-hepatic spread) has a synergistic effect on the underlying hemostatic imbalance (due to liver cirrhosis). This is compounded by other conditions (e.g., obesity), ultimately resulting in an increased thrombotic risk.

The data from a second single-center study [5] confirmed previous reports by Wang et al. wherein among 194 consecutive patients diagnosed with HCC, the incidence of systemic VTE was 6.7%, mostly DVTs (*n* = 7, 63.6%). The median time span from HCC diagnosis to VTE diagnosis was relatively short (4.2 months (range, 0–30.6 months)). Also, 16% of the study population (*n* = 30) had extra-hepatic metastases. Unfortunately, the relative incidence of VTE in this subgroup was not reported though it is entirely plausible that HCC tumor burden (biological and morphological) may influence VTE risk. Interestingly, among patients with long-term follow-up (*n* = 165), those with PVT had a higher rate of systemic VTE versus patients without PVT (11.5% vs. 4.4%; *p* 0.04), possibly suggesting a common mechanism of hemostatic activation (Table 1).

Lesmana et al. [45], in line with our own findings [19], did not observe any increase of VTE risk in neoplastic patients, with HCC evenly distributed among one third of patients with and without DVT (*p* = 0.614). The authors notably limited the discussion of thrombotic complications to DVT, and the presence of DVT was only assessed if patients presented with clinical symptoms (e.g., unilateral leg swelling, leg pain, etc.), possibly underestimating the actual thrombotic risk in HCC patients. Overall, different methods were used for the diagnosis of thromboembolic complications (both portal vein thrombosis and deep vein thrombosis/pulmonary embolism), and different subpopulations have been tested as well (e.g., autoptic samples, liver transplant candidates, decompensated patients etc.). The variety of subpopulations studied and methods used to diagnose thrombotic events might be the cause of different results in terms of frequency of thrombotic complications, making it difficult to compare different analyses. Thus, further prospective studies are urgently needed to better characterize patients who are more at risk for thromboembolic complications and would therefore benefit from early thromboprophylaxis to reduce morbidity and mortality.

The development of VTE in patients with active cancer has several clinical implications [52,57]. Mortality in cancer patients with VTE may be attributable either to the thromboembolic event itself (pulmonary embolism), or to the aggressive behavior of the neoplasm.

Do HCC patients who develop VTE have a greater risk of death than their counterparts with no thrombotic complications? Wang et al. [55] estimated the HR for death in patients with VTE at 3.62 (95% CI = 1.22–10.79, *p* = 0.021 at multivariate analysis), in line with previous data on other types of neoplasms. It remains unclear from a retrospective analysis whether this stemmed solely from the severity of HCC or the thrombotic complication, thus further prospective studies are warranted. Predictably, time to death for all patients who developed PE was within 3 months of the PE diagnosis, as it is an acute and potentially fatal event. Interestingly in the study by Wun et al. [37], the 1-year mortality after liver cancer diagnosis was 76.8%, although only 32% of patients had a metastatic cancer. The authors found a positive correlation between 1-year death rate associated with specific types of cancer and the percentage of cases that developed VTE (*r* = 0.81, *p* = 0.002).

## 3. Pathogenesis of HCC Associated Thrombophilia: The Role of Fibrinogen, Platelets, and Extracellular Vesicles

Cancer patients carry a hypercoagulable state and venous thromboembolic events can be the first clinical sign of cancer [66]. Globally, 20% of all cases of VTE are related to cancer which confers a 4- to 7-fold higher risk of developing VTE [67]. In fact, VTE in cancer patients is associated with plasma hypercoagulability which might be triggered either by the pro-coagulant activity of cancer cells or the host response to cancer. Also, patient-related and treatment-related factors such as chemotherapy, bed rest, infection, and surgery may play a role in increasing VTE risk [68,69,70,71].

**Figure 1:**
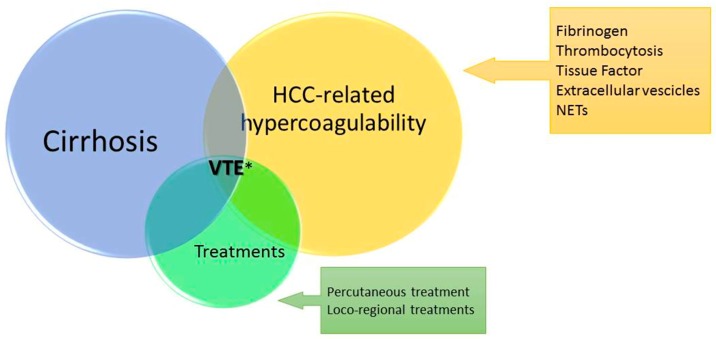
Prothrombotic state in hepatocellular carcinoma. Legends: NETs: neutrophil extracellular traps: VTE: venous thromboembolism: * including portal vein thrombosis.

There is little evidence as it pertains to the presence of such a hypercoagulable state in patients with HCC, and the interplay between hepatoma cells and coagulation homeostasis. This is probably due to the unique biological and clinical context in which HCC arises: the cirrhotic liver. Multiple changes occur in the hemostatic system as a result of deranged liver function. The liver synthesizes most coagulation factors and regulatory proteins that are part of the hemostatic control mechanisms, thus a severe derangement of its function can result in thrombotic complications [72]. Recent studies have suggested multiple and often interrelated mechanisms through which HCC can tip the hemostatic balance towards hypercoagulability (Table 3, Figure 1).

In our experience, fibrinogen levels were slightly higher—though not statistically significant—in patients with HCC than in patients without neoplastic disease [19]. Interestingly, the increase of plasmatic fibrinogen correlated positively with tumor volume, as previously suggested by other studies [73,74]. The increased fibrinogen is attributable to different mechanisms, most notably systemic inflammation [75] though it now appears from recent studies that cancer cells can also synthesize fibrinogen [76]. Moreover, rotational thromboelastometry showed an increase in clot firmness at FIBTEM in patients with HCC, including those in which plasma concentration of fibrinogen was not significantly higher compared to controls, maybe hinting at a qualitative alteration (i.e., polymerization process) as well.

Thrombocytosis has long been associated with different types of cancers such as lung, colorectal, kidney, and glioblastoma [77,78]. Data regarding patients with HCC are sparse and conflicting. Both normal and cancerous hepatocytes are able to synthesize thrombopoietin (TPO), which is the most important growth factor for platelet production and mobilization from the bone marrow [79]. However, the concomitant presence of portal hypertension can inevitably affect the platelet count. In the studies by Hwang et al. [80] and Carr et al. [81], the prevalence of platelet count > 400 × 10^9^ was reported at 2.7% and 9%, respectively. Our findings show a higher platelet count in HCC cirrhotic patients versus HCC-free cirrhotic controls, which was particularly evident in well-compensated patients (Child’s A class) and also correlated with a vital tumor volume greater than 5 cm^3^ [19]. Similarly, Hwang et al. found that the thrombocytosis was correlated with tumour biology and morphological features (level of alpha-foetoprotein and tumor volume) [80]. They also found that HCC patients with thrombocytosis had significantly higher mean serum TPO than those without, and more expression of TPO mRNA were found in tumor tissues than in non-tumor tissues of liver in an HCC patient with thrombocytosis. Bearing this in mind, thrombocytosis in HCC patients may be caused by overproduction of TPO by tumors described mostly in patients with a large tumour burden [78,80,82]. There is still no consensus on whether platelets are hyperactivated in HCC. Indeed, a recent in vivo study by Alkozai et al. failed to detect any differences in the basal and agonist-induced platelet activation between patients with or without HCC [83].

Lastly, expression of tissue factor (TF) by tumor cells may be predictive of systemic VTE in patients with pancreatic and ovarian cancers [84,85]. It has been shown that hepatoma cells can synthesize TF and whether this results in an increased risk for thrombosis is still unclear, hence the need for additional prospective studies [86].

Extracellular vesicles (EVs) are heterogeneous, nano-sized vesicles shed into blood and other body fluids by many cell types, that dispatch a variety of bioactive molecules (e.g., protein, mRNA, miRNA, DNA and lipids) to cellular targets over long and short distances [87]. There are three conventionally recognized EV subtypes that are classified based on their respective size and route of biogenesis [87,88,89]. Two of these are released by cells both constitutively and in response to stimulation, and are characterized as exosomes (30–100 nm diameter) and microvesicles (100–1000 nm diameter, sometimes referred to as ‘microparticles’), though their respective sizes can seldom overlap. Exosomes derive from in-budding of endosomes to form multi-vesicular bodies that fuse with the plasma membrane to release the membrane vesicles into the extracellular space. Microvesicles (MV) form by outward budding of the plasma membrane [90]. MVs and exosomes may play a major role in tumorigenesis, tumor progression, metastasis, and cancer-associated thrombosis [90,91,92,93,94]. Indeed, several groups have described elevated levels of circulating EVs (both MV and exosomes) derived from tumor, host blood cells, and endothelial cells in cancer patients [90,91,92,94,95,96,97,98,99]. The procoagulant potential of MV is mainly due to the exposure of negatively-charged phospholipids (mainly phosphatidylserine) and the vehiculation of TF [90,96]. Exosomes secreted by prostate cancer cells (prostasomes) have been shown to trigger thrombin generation in vitro in a dose-dependent manner as well, and induce lethal pulmonary embolism in mice [98]. Additionally, in a mouse model of breast cancer, tumor-derived exosomes enhanced thrombosis by cooperating in the generation of neutrophil extracellular traps (NETs) [100]. 

Recently, our group considered 65 adult cirrhotic patients (Child: A 21, B 27, C 17), 33 with HCC, 32 without HCC, and 50 controls [101]. Patients with cirrhosis and HCC showed significantly higher median plasma levels of Annexin V-MV (i.e., phosphatidylserine+ MVs), endothelial-derived, platelet-derived, leukocyte-derived MV, as well as TF-bearing MV and thrombomodulin (TM) + MV than cirrhotic patients without HCC and healthy controls. Additionally, HCC-free cirrhotic patients had significantly higher median levels of Annexin V-MV, endothelial-derived, platelet-derived, leukocyte-derived MV, and TM + MV compared to healthy controls. No significant difference was detected for TF + MV between healthy controls and HCC-free cirrhotic patients. These findings confirmed the presence of TF + MV in HCC (as described in other neoplasms) but not in cancer-free cirrhosis. Portal vein thrombosis was detected after 12 months of follow-up in 12 (18%) cirrhotic patients, 8 (25%) with HCC and 4 without HCC (12.5%). The 95th percentile of Annexin V and endothelial-derived MV was calculated in HCC patients who did not develop PVT. Two out of the 8 patients with PVT (25%) and four of the 25 without PVT (16%) had MV levels above this cut-off point, resulting however in a non-significant RR of 1.50 (95% CI 0.40 to 5.69) to develop PVT. Brodsky et al. showed higher levels of Annexin V-MV, endothelial-derived and hepatic cell-derived MV in patients with Hepatitis C and HCC compared to patients with Hepatitis C alone [102]. Table et al. performed a quantitative proteomic analysis on plasmatic MV isolated from 22 HCV-induced cirrhosis patients, 16 HCV-positive HCC patients with underlying cirrhosis, and 18 healthy controls [103]. They mainly found that the proteome composition on plasmatic MV in both disease entities displayed remarkable differences compared to healthy controls, whereas the proteome difference between both diseases was minimal. Particularly, Annexin A2 (ANXA2) was found to be significantly more abundant in HCC compared to both opposed experimental conditions. ANXA2, with its associated proteins, constitutes a key endothelial cell surface entity that regulates two dynamic processes, fibrin balance and angiogenesis, and it is involved both in hemorrhagic and in thrombotic disorders [104]. A recent paper by Julich-Haertel et al. considered 172 patients with liver cancer (HCC or cholangiocarcinoma), 54 with cirrhosis and no liver neoplasia, and 202 control subjects. They showed that the levels of liver tumor-derived MV (marked with ASGPR1 antibody) expressing also Annexin V and EpCAM could significantly discriminate between liver cancer-bearing patients and patients with cirrhosis, allowing for a non-invasive assessment of the presence, and possibly the extent of said cancers in patients with advanced liver diseases [105]. No data regarding the pro-coagulant role of these MV were collected.

Exosomes have been reported to play several key roles in HCC [106]. Particularly, exosomes can induce malignant transformation of liver cells by promoting viral diffusion and inflammation, exchanging oncogenic factors between tumor cells, sustaining tumor growth by neighboring stromal cells, facilitating metastasis, triggering chemoresistance by transmitting long noncoding RNAs, stimulating immune activation/immune evasion, as well as being useful in biomarker detection and therapeutic options. However, there is no reported evidence on the pro-thrombotic potential of exosomes.

In addition to EVs, cancer cells secrete various types of cytokines that modify neutrophil biology, leading to changes in neutrophil counts and state of activation, including the release of NETs [90,107]. Animal models have suggested that NETosis may play a major role in cancer-associated thrombosis. A recent study by van der Windt DJ et al. detected elevated levels of NET markers in the serum of patients with non-alcoholic steatohepatitis (NASH) [108]. Furthermore, NASH-induced cirrhosis in mice showed early neutrophil infiltration and NET formation, followed by an influx of monocyte-derived macrophages, production of inflammatory cytokines, and progression of HCC. Interestingly, inhibiting NET formation via DNAse or knockout mice did not affect the development of a fatty liver though it did alter the ensuing pattern of liver inflammation, which ultimately resulted in decreased tumor growth.

## 4. From Bench-to-Bed Side: Should We Consider Thromboprophylaxis in Cirrhotic Patients with HCC?

Prophylactic anticoagulation is standard practice to prevent potentially life-threatening VTE in cancer patients [109].

The latest guidelines recommend thromboprophylaxis in patients with active cancer throughout hospitalization, whereas there is insufficient data to support routine thromboprophylaxis in patients admitted for minor procedures or short-course chemotherapy infusion. Furthermore, routine thromboprophylaxis is not recommended for ambulatory patients with cancer though it may be indicated in selected high-risk patients [110,111,112].

Indeed, in-hospital VTE prophylaxis should be considered in patients with cirrhosis in the setting of advanced liver disease. Particularly, there have been reports suggesting that thromboprophylaxis for the prevention of PVT could not only reduce thrombotic risk but also improve the outcome in patients with liver disease [113]. The results of a randomized trial in a cohort of patients with advanced cirrhosis showed that anticoagulant treatment with low molecular weight heparin (LMWH) was safe (no relevant side effects or hemorrhagic events were reported) and effective, significantly reducing the risk of PVT development and liver decompensation, markedly improving liver function and Child–Pugh score, and improving overall survival [18].

Prevention of PVT may not be the only event responsible for all these beneficial effects: heparin alone may slow the progression of liver disease by virtue of anticoagulation improving intestinal microcirculation, which in turn reduces bacterial translocation and thus the risk of bacterial infections. If prothrombotic states accelerate liver fibrosis and coagulation proteins activate hepatic stellate cells to promote fibrosis, then conversely, anticoagulation may reverse hepatic fibrosis. In fact, findings from several animal studies support this hypothesis; warfarin and thrombin antagonists have shown anti-fibrotic properties in a carbon tetrachloride mouse model of liver fibrosis [114]; Rivaroxaban, an oral direct-acting FXa inhibitor, has proven more effective than direct thrombin inhibition in suppressing fibrosis in a thioacetamide mouse model of liver fibrosis [115]; prolonged administration of enoxaparin in a rat model of cirrhosis (induced using carbon tetrachloride or thioacetamide) resulted in the improvement of both portal hypertension and liver fibrosis, presumably by potentiating fibrosis regression and ultimately decreasing portal pressure [116]. Alongside the clinical response to warfarin therapy in pulmonary fibrosis, these results provide a rationale for testing anticoagulants as a treatment for liver fibrosis wherein the cause of the underlying liver disease cannot be removed.

As mentioned earlier, we studied a group of cirrhotic patients with HCC and found that thrombocytosis was more evident in Child’s A cirrhosis [19], hence the potential benefit of thromboprophylaxis. Moreover, a recent paper on patients undergoing liver resection for primary liver cancer in China and treated with prophylactic subcutaneous injection of LMWH 2–7 days after surgery, reported an estimated prevalence of VTE in the treated group at 0.85% (1/117), which was lower than that observed in the control group (13.79%) (16/116); the difference was statistically significant (*p* < 0.05) [117]. No bleeding complications were reported.

To the best of our knowledge, there is currently no evidence regarding the use of prophylactic anticoagulation to prevent PVT or VTE in HCC. The current score system to help determine whether to initiate thromboprophylaxis in outpatients (Khorana score) [118] deems cancer high and very high thrombotic risk. As we mentioned previously, HCC might be associated with an intermediate thrombotic risk, according to current data. However, as no prospective studies including patients with only liver cancer (with and without cirrhosis) have been conducted to evaluate the incidence of thrombotic complications, it is impossible at this point to infer the VTE-associated mortality.

The only evidence we currently have is that liver cancer is associated with an intermediate risk to develop thrombotic complications (both local and systemic) and thromboprophylaxis in patients with cirrhosis has proven relatively safe. In fact, bearing in mind the increased thrombotic risk associated with HCC, it is natural for liver specialists to have safety concerns regarding the bleeding risk related to anticoagulant drugs when used in people with advanced liver disease, especially in presence of significant thrombocytopenia and/or varices. Studies of VTE prophylaxis in cirrhotic patients have demonstrated no significant increase in the risk of bleeding with anticoagulation [119,120]. Reichert and colleagues demonstrated that INR > 1.5 correlated with a higher risk of bleeding, though it only applied to minor bleeding [121]. Stratification by type of anticoagulation has demonstrated that unfractionated heparin results in a higher bleeding risk than LMWH in cirrhotic patients. Fittingly, a prophylactic dose of enoxaparin (4000 IU daily) to prevent PVT in patients with Child–Pugh B/C cirrhosis had no bearing on the risk of bleeding events when heparin was administered for a period of 48 weeks compared to a matched control group of patients treated with placebo alone [18]. Cirrhotic patients are likely more sensitive to unfractionated heparin [122] and therapeutic doses of this type of heparin also resulted in a significant drop in hemoglobin and platelet counts in these patients [123].

With conventional therapy, cancer patients have higher rates of recurrent VTE and 2- to 6-fold greater risk of anticoagulant-related major bleeding (an absolute incidence of 3–9% in the first 6 months of treatment) compared with cancer-free patients, but current data suggest that the clinical presentation and course of anticoagulant-related major bleedings are not more severe in cancer patients [124]. Nevertheless, the reluctance to use anticoagulant therapy in anticipation of serious bleeding and the limitations of the currently recommended first-line treatments with LMWH or, alternatively, vitamin K antagonists (VKA) often result in significant underuse of thromboprophylaxis in cancer patients [67,125]. Non-VKA oral anticoagulants (NOACs)—direct thrombin inhibitor dabigatran and direct inhibitors of activated FX rivaroxaban, apixaban, and edoxaban—are increasingly investigated as viable options for many unmet needs in the treatment of cancer-associated thrombosis [126,127]. At present, there is no data specifically evaluating the hemorrhagic risk in cirrhotic patients with HCC and studies are needed to determine in which cirrhotic patients with HCC, and when, the benefit of anticoagulation exceeds the risk.

## 5. Conclusions

In conclusion, cirrhotic patients with hepatocellular carcinoma may exhibit hypercoagulability, with its main determinant being the increased fibrinogen concentration/polymerization and thrombocytosis related to cancer. From a pathophysiological point of view, these patients have an increased risk of thrombotic complications for two main reasons. Firstly, the unstable hemostatic balance of liver cirrhosis, which can be easily tipped towards hypercoagulability. Secondly, the presence of liver cancer, and namely, cirrhotic patients with HCC showed higher levels of TF-expressing microvesicles than HCC-free cirrhotic patients. Unfortunately, it is still unclear which patients carry a higher risk of developing thrombotic complications and there are no prospective studies on this matter. Given the increased mortality and morbidity associated with thrombotic complications, it is of the utmost importance to assess which patients have a higher risk of thrombosis (portal vein thrombosis and systemic thromboembolic complications) and would therefore most likely benefit from early thromboprophylaxis. Some studies suggest that global coagulation tests could be useful in that regard but robust data are sorely lacking. The decision whether to use anticoagulation in cirrhosis patients requires a careful assessment of its perceived risks and benefits. Current guidelines do not recognize the thromboembolic risk associated with chronic liver disease, and do not make specific recommendations on the prophylaxis or treatment of thromboembolic disease in this unique population. While waiting for further prospective studies that can definitively answer that question, the interim suggestion is that thromboprophylaxis should be considered on a case-by-case basis for cirrhotic patients, based on risk factor assessment. More precisely, patient-related variables pertaining to liver cancers and its effects (mostly tumor burden, fibrinogen concentration, platelet count, presence of hypercoagulability at viscoelastic tests, TF+MVs), as well as the clinical scenario (cirrhotic patient with HCC waiting for liver transplantation, invasive procedures) should be taken into consideration to ascertain in which cases the benefits of thromboprophylaxis exceed the risk of anticoagulation.

## Figures and Tables

**Table 1:** Thromboembolic complications in cirrhotic patients with hepatocellular carcinoma.

Author, Years [Ref]	Type of Study	Population	Method for Thrombosis Diagnosis	Incidence of Thrombotic Complications	Statistical Significance
**Portal Vein Thrombosis (PVT)**					
Nonami, 1992 [32]	Retrospective, single center	- 87 patients with cirrhosis and HCC - 401 patients with post-necrotic liver cirrhosis Autoptic population	Examinations of excised livers at the time of LT	30/87 (34.8%) vs. 63/401 (15.7%)	NA
Davidson, 1994 [33]	Prospective, single center	- 22 patients with cirrhosis and HCC - 110 patients non-HCC cirrhosis LT candidates	Operative finding at the time of LT	6/22 (27.3%) vs. 10/110 (9.1%)	<0.05
Ravaioli, 2011 [34]	Retrospective, single center	- 282 patients with cirrhosis and HCC LT candidates	Operative finding at the time of LT	37/282 (11%)	HCC significantly associated with PVT risk at multivariate analysis (HR: 1.81; *p* < 0.05)
Zanetto, 2017 [19]	Prospective, single center (1-year f-up)	- 41 patients with cirrhosis and HCC - 35 patients with non-HCC cirrhosis Both compensated and decompensated patients	Splanchnic Doppler ultrasound and subsequently characterized by CT/MRI	10/41 (24.4%) vs. 4/35 (11.4%)	0.05
**Extra-Splanchnic Thromboembolic Complications**					
Levitan, 1999 [36]	Retrospective, US Medicare data	- 22,938 patients with discharge diagnosis of liver cancer 1988–1990	Subsequent discharge diagnosis of DVT/PE	121/22,938 = 69 per 10,00 patients	Intermediate risk (same risk of lung cancer)
Wun, 2009 [37]	Retrospective, California Discharge data	- 2312 patients with discharge diagnosis of liver cancer 1993–1999	Subsequent discharge codes for VTE	1-year cumulative incidence 1.7% 1-year rate 4.1%/100 patients-years	- Intermediate risk (same risk as lung cancer) - Correlation between 1-year death rate and VTE incidence
Cronin-Fenton, 2010 [38]	Retrospective, Danish medical record data	- 550 patients with diagnosis of liver cancer 1997–2005 - general population cohort	Subsequent diagnosis code for VTE	6/550 (1.1%) vs 11/2746 (0.4%)	High risk
Connolly, 2008 [5]	Retrospective, single center	- 194 consecutive patients with cirrhosis and HCC	Splanchnic Doppler ultrasound and CT/MRI at the time of LT; VTE not specified	6.7% Most of VTEs (7, 63%) were DVTs	PVT patients had a higher rate of systemic VTE vs non-PVT patients
Lesmana, 2010 [45]	Case control, single center	- 87 patients with cirrhosis and HCC- 169 cirrhotic patients without HCC	Lower limb Doppler ultrasound in the presence of clinical symptoms	4/87 (4.6%) vs 8/169 (4.7%) *	0.6
Wang, 2018 [55]	Retrospective, single center	- 270 consecutive patients with cirrhosis and HCC Both compensated and decompensated patients	Lower limb Doppler ultrasound, thoracic CT scan	6% (2-years cumulative incidence) 12/16 (75%) early VTE	>3 hepatic lesions vs single lesion (HR = 3.6, *p* < 0.05); multi-organ extra-hepatic metastasis (HR = 12; *p* < 0.05) significantly associated with VTE risk at multivariate analysis

NA: not available; DVT: deep vein thrombosis; PE: pulmonary embolism; HCC: hepatocellular carcinoma; LT: liver transplant; VTE: venous thromboembolism; PVT: portal vein thrombosis; HR: hazard ratio; DVT: deep vein thrombosis; CT: computed tomography; MRI: magnetic resonance imaging. * Only cases of deep vein thrombosis were included in the analysis.

**Table 2:** Extra-splanchnic thromboembolic complications in patients with cirrhosis.

Author, Year [Ref]	Type of Study	Patients with Liver Disease (*n*)	VTE Prevalence (%)	DVT Prevalence (%)	PE Prevalence (%)
Northup, 2006 [39]	Case control	21,000	0.5	0.35	0.1
Garcia Fuster, 2008 [43]	Retrospective	2074	0.8	0.5	0.3
Gulley, 2008 [47]	Case control	963	-	1.8	0.9
Lesmana, 2010 [45]	Retrospective	256	4.7	4.7	-
Dabbagh, 2010 [48]	Retrospective	190	6.3	-	-
Wu, 2010 [58]	Retrospective	241,626 ^ 408,253 ^§^	0.81 0.82	-	-
Aldawood, 2011 [41]	Retrospective	226	2.7	2.7	-
Saleh, 2011 [59]	Retrospective	4,565,000	0.9	0.6	0.2
Ali, 2011 [42]	Retrospective	449,798	1.8	1	0.9
Girleanu, 2012 [44]	Retrospective	3108	2.5 *	0.99	
Kohsaka, 2012 [60]	Retrospective	719	1.4	0.8	0.8
Shah, 2012 [46]	Retrospective	85	-	7	-
Al-Dorzi, 2013 [40]	Retrospective	75	-	2.7	-
Walsh, 2013 [61]	Retrospective	2606	1	-	-
Ponziani, 2013 [62]	Retrospective	10,359	0.3	0.1	0.2
Bogari, 2014 [63]	Retrospective	163	11	-	-
Shatzel, 2015 [64]	Retrospective	233 ^>^	2.4	-	-
Yang, 2018 [65]	Retrospective	108 ^>^	0.9	-	-

^ Patients with compensated liver cirrhosis. ^§^ Patients with decompensated liver cirrhosis. only patients with non-alcoholic liver cirrhosis (patients with alcoholic liver diseases presented with a lower prevalence of VTE, 0.6%). * Including patients with portal vein thrombosis. ^>^ Including only patients without thromboprophylaxis.

**Table 3:** HCC-associated hypercoagulability.

**Known Molecular Risk Factors**		
**Molecular pathway**	**Mechanism**	**Ref**
Increased fibrinogen levels/activity	-↑systemic inflammation -↑synthesis by cancer cells	[19,73,74,75]
Thrombocytosis	- overproduction of thrombopoietin by cancerous hepatocytes	[79]
Tissue Factor (TF)	-↑synthesis by hepatoma cells	[86]
**Novel Molecular Risk Factors**		
Extracellular microvesicles (MVs)	-↑Annexin V, endothelial-derived, platelet-derived, leukocyte-derived, TF-bearing and thrombomodulin+MVs in HCC and cirrhosis compared to HCC-free cirrhosis and healthy controls -↑Annexin V, endothelial-derived and hepatic-derived MVs in HCC and Hepatitis C compared to Hepatitis C alone	[101,102,103]
Exosomes	- role in tumorigenesis and metastatization	[106]
NETs	- NET formation in livers from NASH induced-mice, influx of monocyte-derived macrophages, inflammatory cytokines, and progression of HCC	[108]

HCC, hepatocellular carcinoma; MVs, microvesiscles; TF, tissue factor; NETs, neutrophil extracellular traps; NASH, non-alcoholic steatohepatitis; ↑, increased.
